# Risk factors analysis for clinical symptoms of prenatally diagnosed choledochal cysts: a retrospective study

**DOI:** 10.1186/s12893-023-02115-2

**Published:** 2023-08-04

**Authors:** Dan Yang, Long Li, Mei Diao, Xianghui Xie, Anxiao Ming, Ruyue Gao, Yu Tian

**Affiliations:** 1 Department of Pediatric Surgery, Children’s Hospital Capital Institute of Pediatrics, Chinese Academy of Medical Sciences & Peking Union Medical College, Research Unit of Minimally Invasive Pediatric Surgery on Diagnosis and Treatment, Chinese Academy of Medical Sciences 2021RU015, 100020 Beijing, China; 2 Department of Pediatric Surgery, Tsinghua University Affiliated Beijing Tsinghua Changgung Hospital, 102218 Beijing, China

**Keywords:** Choledochal cyst, Symptoms, Prenatal diagnosis

## Abstract

**Background:**

This study aimed to screen the impact factors for clinical symptoms of prenatally diagnosed choledochal cysts (CDCs), to warn about the occurrence of clinical symptoms and the timing of surgery.

**Methods:**

Medical records of patients with prenatally diagnosed CDCs admitted to our hospital from April 2013 to April 2018 were retrospectively reviewed. Fetal hilar or abdominal cysts were found by prenatal ultrasonogram. All patients underwent laparoscopic cyst excision and hepaticojejunostomy in our center. Univariate analysis and multivariate logistic regression analysis were performed to screen the factors related to clinical symptoms intimately.

**Results:**

Two hundred eighteen cases were included. One hundred thirty-four patients (134/218, 61.5%) presented clinical symptoms before surgery. The results of univariate analysis showed that patients with clinical symptoms had earlier time of prenatal diagnosis (*P* = 0.002), higher values of GGT, TBIL, DBIL (*P* < 0.001, *P* < 0.001, *P* < 0.001, respectively) and larger maximum diameter of cyst before surgery (*P* = 0.012). Multivariate logistic regression analysis suggested that the time of prenatal diagnosis (*P* = 0.001, *OR* = 0.898, 95% *CI*: 0.845 ~ 0.955) and the GGT value within one week of life (*P* = 0.028, *OR* = 1.002, 95% *CI*: 1.000 ~ 1.003) were independent influencing factors for symptoms.

**Conclusions:**

For children with prenatally diagnosed CDCs, approximately 2/3 patients presented noticeable clinical symptoms before surgery. The time of prenatal diagnosis and the GGT value within 1 week of life were independent impact factors for the occurrence of clinical symptoms.

## Introduction

Choledochal cyst (CDC) is a common biliary malformation characterized by common bile duct dilatation with or without intrahepatic bile duct dilatation. It is more common in Asian countries and affects more females. Laparoscopic cyst excision and Roux-Y hepaticojejunostomy are the main methods for the treatment of CDC [1]. Delayed diagnosis and treatment may cause the occurrence of clinical symptoms before surgery, at which time the surgical effect is not good. With the development of prenatal ultrasound diagnosis technology, the diagnosis of CDC has gradually shifted from postnatal to prenatal [2, 3]. For children with prenatally diagnosed CDCs, clinical symptoms are one of the influencing factors that determine the optimal time for surgery [4]. However, no prior reports with a large pediatric series have focused on the risk factors of clinical symptoms after birth. The impact factors for symptoms are unclear, and the operation opportunity is controversial. Therefore, the purpose of this study was to screen the impact factors of clinical symptoms in children with prenatally diagnosed CDCs.

## Materials and methods

### Study population and data collection

Two hundred sixty-two patients with CDCs detected on prenatal ultrasonogram were admitted to our hospital from April 2013 to April 2018. Twenty-three cases were excluded because of incomplete clinical data or previous operations in other hospitals. Nineteen cases were excluded due to a modified diagnosis confirmed by intraoperative laparoscopy and cholangiography, with 15 cases of cystic biliary atresia, 1 case of omental cyst, 1 case of ovarian cyst, 1 case of intestinal duplication cyst and 1 case of duodenal atresia. Two cases of Caroli disease were excluded. Prenatal ultrasonogram when the cyst was first detected, abdominal ultrasound and laboratory tests within 1 week of life, abdominal ultrasound, computed tomography (CT), magnetic resonance cholangiopancreatography (MRCP) and laboratory tests before surgery, as well as intraoperative cholangiogram were collected. Prenatal ultrasonogram was completed in tertiary maternity hospitals, and all mothers of patients underwent prenatal ultrasonogram at similar pregnancy stages. All patients underwent laparoscopic cyst excision and hepaticojejunostomy by the same group of experienced physicians in our center.

Patients were divided into two groups according to whether there were clinical symptoms after birth: symptomatic group (n = 134, 61.5%), asymptomatic group (n = 84, 38.5%).

### Ethics

This study was conducted in accordance with the principles of the Declaration of Helsinki, and this study was approved by the Ethics Committee of Capital Institute of Pediatrics with waiver of informed consent (shell2022047). The requirement for informed consent was waived by the Ethics Committee of Capital Institute of Pediatrics because of the retrospective nature of the study.

### Statistical analysis

SPSS 26.0 statistical software was used for data analysis. Categorical data was described as case number and percentage, chi-square test or Fisher’s exact test was used to compare the proportion of data between two groups. Nonnormally distributed data was described as *M* (*P*_25_, *P*_75_), Mann–Whitney U test was used for comparison between the two groups. The value of tolerance and variance inflation factor (VIF) were used to evaluate the multicollinearity diagnosis of factors. Multivariate Logistic regression analysis was performed to screen the independent risk factors of clinical symptoms.

## Results

### Clinical data

A total of 218 patients were included in the study. Detailed information on the 218 CDC cases is shown in Table 1, and these cases included 59 males and 159 females (ratio of 1:2.7). The time of prenatal diagnosis ranged from 12 to 40 weeks of gestation, with a median time of 24 (20.5, 28) weeks. The age at surgery ranged from 3 days to 55 months, with a median age of 1.8 (0.9, 4.8) months. A total of 202 patients (92.7%) were younger than 1 year old, and 53 patients (24.3%) were newborns. The weight at surgery ranged from 2.13 to 15.5 kg, with a median weight of 5.0 (3.8, 7.0) kg. Todani classification [5] analysis showed that 117 CDCs were type I, 101 CDCs were type IV. No type II or III CDC was observed in this study. Specific classifications of type I and IV are shown in Table 1.

**Table 1:** Clinical data of prenatally diagnosed CDCs

	Prenatally diagnosed CDCs (n = 218)
Gender (M/F)	59/159 (1:2.7)
Prenatal diagnostic time (weeks)	24 (20.5, 28)
Age at su**rge**ry(m)	1.8 (0.9, 4.8)
Weight at surgery (kg)	5.0 (3.8, 7.0)
Todani	
Ia	111, 50.9%
Ib	4, 1.8%
Ic	2, 0.9%
IVa	100, 45.9%
IVb	1, 0.5%

Clinical data of prenatally diagnosed CDCs. Nonnormally distributed data was described as *M* (*P*_25_, *P*_75_). Categorical data was described as case number and percentage

### Clinical symptoms

A total of 134 cases presented clinical symptoms after birth. The results showed that 80 cases were yellow staining of the skin or sclera, 40 cases were white stool or light-colored stool (white stool or light-colored stool were predominant, with or without yellow staining of the skin or sclera), and 14 cases were spitting milk in infants or vomiting in children. Of these patients with clinical symptoms, 93 patients developed symptoms within 3 months of life, 19 patients developed symptoms from 3 to 6 months, and 22 patients developed symptoms after 6 months (Table 2).

**Table 2:** Clinical symptoms of prenatally diagnosed CDCs

	Symptomatic CDCs (n = 134)
Type	
yellow staining	80, 59.7%
White or light-colored stool	40, 29.9%
spitting milk or Vomiting	14, 10.4%
Time of occurrence	
< 3 months	93, 69.4%
3–6 months	19, 14.2%
> 6 months	22, 16.4%

Type and the time of occurrence for clinical symptoms in children with prenatally diagnosed CDCs. Categorical data was described as case number and percentage.

### Univariate analysis for clinical symptoms of prenatally diagnosed CDCs

#### The time of prenatal diagnosis

The prenatal diagnostic time of symptomatic group was 24 (20, 25) gestational weeks, and that of asymptomatic group was 24 (23, 28) gestational weeks. Analysis suggested that the prenatal diagnostic time of asymptomatic group was smaller than that of asymptomatic group, with a statistically significant difference (*P* = 0.002).

#### Hepatic function within the first week of life

Postnatal hepatic function was measured within 1 week of life by alanine aminotransferase (ALT), aspartate aminotransferase (AST), total bilirubin (TBIL), direct bilirubin (DBIL) and γ- glutamyl transpeptidase (GGT) values. The GGT, TBIL and DBIL values of the symptomatic group were statistically higher than those of the asymptomatic group (*P* < 0.001, *P* < 0.001, *P* < 0.001, respectively). However, no significant difference in the ALT or AST values was observed between the two groups (*P* = 0.356, *P* = 0.752) (Table 3).

**Table 3:** Comparison of hepatic function within the first week of life

	Symptomatic group (n = 134)	Asymptomatic group (n = 84)	*P*
ALT (U/L)	21.5 (13.7, 43.9)	26.0 (17.0, 37.4)	0.356
AST (U/L)	41.8 (29.9, 61.3)	42.2 (32.9, 52.8)	0.752
GGT (U/L)	163.0 (89.3, 387.5)	70.0 (25.0, 122.1)	0.000^*^
TBIL (umol/L)	75.6 (28.5, 160.2)	21.1 (9.3, 71.1)	0.000^*^
DBIL (umol/L)	11.5 (5.9, 26.2)	4.8 (2.2, 11.6)	0.000^*^

Comparison of hepatic function within the first week of life between the two groups. Nonnormally distributed data was described as *M* (*P*_25_, *P*_75_)

*P value < 0.05

#### Hepatic function before surgery

Hepatic function within one week before surgery were tested in all patients. Analysis showed that there was no significant difference in ALT, AST, GGT, TBIL or DBIL levels between the two groups (*P* > 0.05) (Table 4).

**Table 4:** Comparison of hepatic function before surgery

	Symptomatic group (n = 134)	Asymptomatic group (n = 84)	*P*
ALT (U/L)	24.3 (17.3, 43.1)	24.0 (17.7, 45.7)	0.686
AST (U/L)	43.3 (35.2, 58.5)	43.9 (33.8, 58.9)	0.697
GGT (U/L)	106.6 (25.9, 381.7)	132.0 (62.0, 351.1)	0.235
TBIL (umol/L)	30.4 (9.3, 95.3)	34.1 (15.1, 114.5)	0.220
DBIL (umol/L)	6.8 (2.0, 17.7)	8.1 (4.0, 26.1)	0.159

Comparison of hepatic function within one week before surgery between the two groups. Nonnormally distributed data was described as *M* (*P*_25_, *P*_75_)

#### The size of CDCs

**Fig. 1:**
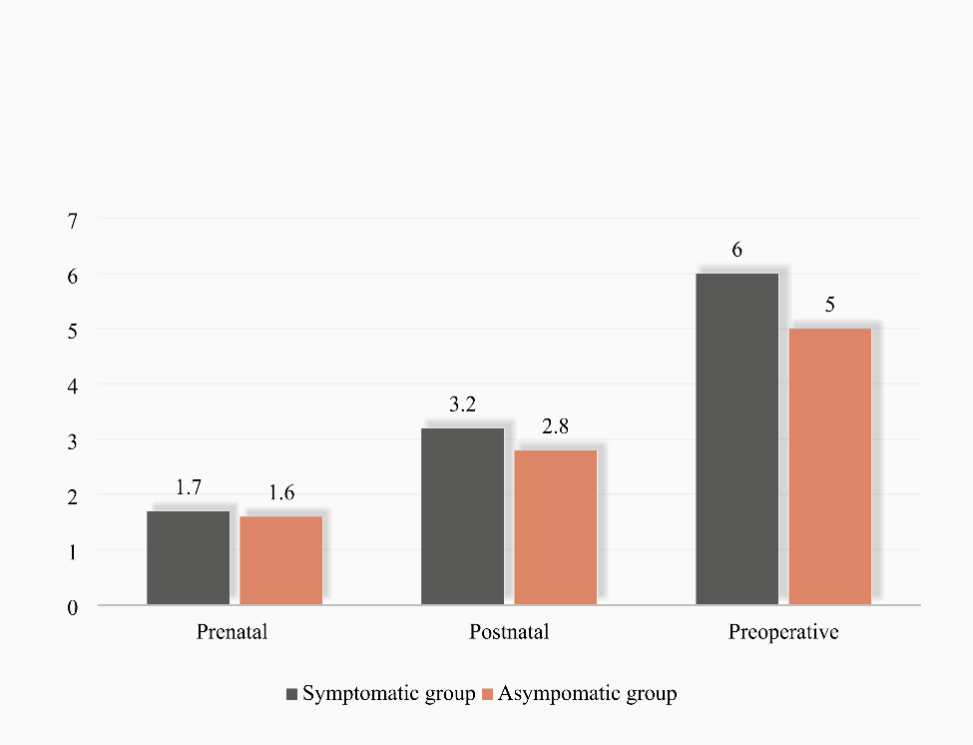
Comparison of the cyst sizes. Comparison of the cyst sizes of prenatal diagnosis, postnatal and before surgery. There was no statistical difference in the prenatal cyst size or postnatal cyst size between the two groups. The cyst size before surgery in symptomatic group were larger than that of asymptomatic group

Hilar or abdominal cysts were detected by prenatal ultrasonography in all patients during fetal life, and abdominal ultrasound was reviewed within one week after birth and before surgery. By comparing the maximum cyst diameter of the two groups at prenatal diagnosis, within one week after birth and before surgery, it was found that the maximum diameter of cyst before surgery of the symptomatic group was larger than that of the asymptomatic group (*P* = 0.012). No significant difference in the maximum diameter of cyst at prenatal diagnosis or within the first week of life was found between the two groups (*P* = 0.859, *P* = 0.115) (Table 5; Fig. 1).

**Table 5:** Comparison of the cyst sizes

	Symptomatic group (n = 134)	Asymptomatic group (n = 84)	*P*
Prenatal	1.7 (1.2, 2.1)	1.6 (1.2, 2.1)	0.859
Postnatal	3.2 (2.3, 4.3)	2.8 (2.1, 4.0)	0.115
Preoperative	6.0 (4.5, 7.9)	5.0 (4.0, 6.3)	0.012^*^

Comparison of the maximum diameter of cyst at prenatal diagnosis, one week after birth and before surgery between the two groups. Nonnormally distributed data was described as *M* (*P*_25_, *P*_75_)

*P value < 0.05

#### Multivariate analysis of clinical symptoms of prenatally diagnosed CDCs

The significant factors (*P* < 0.05) in univariate analysis including prenatal diagnostic time, the maximum diameter of cyst before surgery, the GGT, TBIL and DBIL values within the first week of life. The results of multicollinearity diagnosis manifested that all the values of tolerance were between o.1 to 1 and the VIF bellowed 10, mean there is no collinearity, and the model was stable. Therefore, the multivariate binary Logistic regression analysis was available. Multivariate binary Logistic regression analysis showed that the time of prenatal diagnosis (*P* = 0.001, *OR* = 0.898, 95%*CI*: 0.845–0.955) and the GGT value within the first week of life (*P* = 0.028, *OR* = 1.002, 95%*CI*: 1.000 ~ 1.003) were independent impact factors for clinical symptoms in children with prenatally diagnosed CDCs. The maximum diameter of cyst before surgery, TBIL and DBIL levels within the first week of life were not independent impact factors for symptoms (*P* = 0.424, *P* = 0.085, *P* = 0.440, respectively). (Table 6)

**Table 6:** Multivariate analysis of clinical symptoms

	*B*	SE	Wald*x*^*2*^	*P*	*OR*	95%*CI*
The time of Prenatal diagnosis	-0.107	0.031	11.869	0.001*	0.898	0.845, 0.955
The maximum diameter of cyst	0.079	0.099	0.638	0.424	1.082	0.891, 1.314
GGT	0.002	0.001	4.825	0.028*	1.002	1.000, 1.003
TBIL	0.004	0.002	2.970	0.085	1.004	0.999, 1.008
DBIL	0.008	0.010	0.597	0.440	1.008	0.988, 1.028

Multivariate analysis of clinical symptoms in prenatally diagnosed CDCs. Maximum cyst diameter means the maximum cyst diameter within one week before surgery. GGT、TBIL and DBIL means the GGT、TBIL and DBIL value within the first week of life

*P value < 0.05

## Discussion

The clinical symptoms of patients with CDCs can occur at any age and are mainly manifested as abdominal pain, jaundice, and abdominal mass. However, as prenatal diagnosis techniques have improved, more CDCs have been detected in the fetal period, even as early as 15 weeks of gestation. Prenatal diagnosis is significant for the early diagnosis and treatment of CDC, as well as the improvement of prognosis [3, 6]. In this study, two cases were diagnosed in the first trimester of gestation, the rest were diagnosed in the second or third trimester, and the earliest prenatal diagnosis time was 12 weeks of gestation. Univariate analysis showed that patients with clinical symptoms had earlier prenatal diagnosis time than patients without clinical symptoms. Among the children admitted to our hospital with suspected prenatally diagnosed CDCs, all of them were detected hilar or abdominal cyst by prenatal ultrasonogram in the fetal period. However, careful differential diagnosis before and during surgery are still needed, especially cystic biliary atresia (CBA), is similar to CDC in clinical symptoms and ultrasonic manifestations, and requires timely surgical treatment [7]. In this group, laparoscopic exploration and intraoperative cholangiography were performed in all cases, some of them were excluded due to modified diagnosis, such as CBA, omental cyst, ovarian cyst, duodenal duplication, etc.

Most of CDCs are diagnosed before the patients reaches 10 years of age, and most patients may present obvious clinical symptoms [8]. The type of clinical symptoms mainly depends on the age at which they occur. In children less than 6 months old, the clinical manifestations are mainly jaundice, which may be caused by extrahepatic cysts that may lead to complete obstruction of the biliary tract. In contrast, clinical symptoms in adults are usually dominated by abdominal pain. Some patients may develop symptoms of cholangitis or pancreatitis that require hospitalization [9]. All the patients in this group were prenatally diagnosed with CDC, and the age at surgery was young. About 61% of the patients presented obvious clinical symptoms before surgery, which was consistent with previous reports [10, 11]. Of the patients with clinical symptoms, 69.4% (93/134) developed symptoms within 3 months of life, and 14.2% (19/134) developed symptoms from 3 to 6 months. That means, the incidence of clinical symptoms was high within 6 months after birth, especially within 3 months. Differing from older patients, who usually have abdominal pain as the main clinical symptom, the main clinical symptoms in our study were symptoms of biliary obstruction, indicating that the biliary tract obstruction was severe in children with prenatally diagnosed CDCs.

The number of prenatally diagnosed CDCs has increased since the first case reported in 1980. Prenatal diagnosis may lead to early surgical treatment. However, the optimal operation opportunity remains controversial, especially for asymptomatic patients [12]. Usually, the occurrence of clinical symptoms is a recognized indication for surgery in children with prenatally diagnosed CDCs. For asymptomatic patients, some researchers suggest that surgery should be performed at least 3 months or 3–6 months after birth [13, 14], while some researchers believe that the surgical time should be as early as possible, especially before severe clinical symptoms present [3]. Hanna et al. ‘s study showed that, compared to patients who undergo CDC excision during the same admission for CDC-related symptoms, elective CDC excision is associated with shortened hospital stay and decreased opioid use among children [15]. A prospective cohort study by Diao et al. suggested that for asymptomatic children with prenatally diagnosed CDCs, the degree of liver fibrosis was higher in children with late surgery (> 1 month) than in children with early surgery (≤ 1 month), and the postoperative recovery of liver function was significantly delayed in children with high degree of liver fibrosis. Therefore, they suggest that asymptomatic patients should undergo operation early even in the neonatal period [16]. In this study, the GGT, TBIL and DBIL values (within 1 week after birth) of patients with clinical symptoms were significantly higher than those without clinical symptoms. Most of the symptoms occurred within 3 months of life. Therefore, for children with prenatally diagnosed CDCs, a part of them may develop clinical symptoms if surgery is delayed to 3 or 6 months of life, which may lead to liver function damage. Multivariate analysis was performed based on the results of univariate analysis. The results of multivariate analysis showed that the time of prenatal diagnosis and the GGT value within 1 week of life were independent influencing factors for the occurrence of clinical symptoms after birth. The time of prenatal diagnosis was a protective factor, that is, the risk of clinical symptoms occurring decreased to 0.896 times for every 1 week increase of prenatal diagnostic time. The GGT value within 1 week after birth was a risk factor, which means the risk of clinical symptoms occurring increased to 1.002 times for every 1U/L increase in GGT value. 

We also studied the relationship between the cyst size and clinical symptoms. The results suggested that the size of cysts in the symptomatic group increases faster than that in asymptomatic group, and the maximum diameter of cyst before surgery was significantly larger in symptomatic patients than in asymptomatic patients. These indicated that although it is not an independent influencing factor, the cyst size may affect the occurrence of clinical symptoms. Cochran et al. analyzed the data of 23 patients with prenatally diagnosed CDCs at four fetal centers in the United States. They found that for newborns, a cyst size ≥ 4.5 cm on initial postnatal ultrasound were associated with the occurrence of symptoms within one month of life [17]. Guan et al. conducted a retrospective analysis of the data of 125 patients with prenatally diagnosed CDCs in a single center in China. They observed that the length of the cyst > 5.2 cm and the width of the cyst > 4.1 cm indicate the possibility of clinical symptoms occurring [18]. Therefore, for patients with the large size of cyst, clinical symptoms may present before surgery, the operation should be performed early.

Limitations: First, this study involves single-center research, and data from more centers are needed to verify our results. Second, this work is a retrospective study, and a large prospective trial will be needed to summarize the clinical experience.

## Conclusions

For children with prenatally diagnosed CDCs, approximately 2/3 patients presented noticeable clinical symptoms before surgery. The time of prenatal diagnosis and the GGT value within 1 week of life were related to the occurrence of clinical symptoms intimately, they can serve as indicators for selecting surgical timing.

## Data Availability

The datasets generated and/or analyzed during the current study are not publicly available due to the protection of personal privacy but are available from the corresponding author on reasonable request.

## References

[1] Friedmacher F, Ford KE, Davenport M (2019). Choledochal malformations: global research, scientific advances and key controversies. Pediatr Surg Int.

[2] Howell CG, Templeton JM, Weiner S, Glassman M, Betts JM, Witzleben CL (1983). Antenatal diagnosis and early surgery for choledochal cyst. J Pediatr Surg.

[3] Weng R, Hu W, Cai S, Guo X, Luo Q (2016). Prenatal diagnosis and prognosis assessment of congenital choledochal cyst in 21 cases. J Obstet Gynaecol.

[4] Lugo-Vicente HL (1995). Prenatally diagnosed choledochal cysts: observation or early surgery?. J Pediatr Surg.

[5] Todani T, Watanabe Y, Toki A, Morotomi Y (2003). Classification of congenital biliary cystic disease: special reference to type ic and IVA cysts with primary ductal stricture. J Hepatobiliary Pancreat Surg.

[6] Cherqaoui A, Haddad M, Roman C, Gorincour G, Marti JY, Bonnard A (2012). Management of choledochal cyst: evolution with antenatal diagnosis and laparoscopic approach. J Minim Access Surg.

[7] Yu P, Dong N, Pan YK, Li L (2022). Comparison between cystic biliary atresia and choledochal cyst: a clinical controlled study. Pediatr Surg Int.

[8] Shi LB, Peng SY, Meng XK, Peng CH, Liu YB, Chen XP (2001). Diagnosis and treatment of congenital choledochal cyst: 20 years’ experience in China. World J Gastroenterol.

[9] de Vries JS, de Vries S, Aronson DC, Bosman DK, Rauws EA, Bosma A (2002). Choledochal cysts: age of presentation, symptoms, and late complications related to Todani’s classification. J Pediatr Surg.

[10] Tanaka H, Sasaki H, Wada M, Sato T, Kazama T, Nishi K (2015). Postnatal management of prenatally diagnosed biliary cystic malformation. J Pediatr Surg.

[11] Urushihara N, Fukumoto K, Yamoto M, Miyake H, Takahashi T, Nomura A (2018). Characteristics, management, and outcomes of congenital biliary dilatation in neonates and early infants: a 20-year, single-institution study. J Hepatobiliary Pancreat Sci.

[12] Foo DC, Wong KK, Lan LC, Tam PK (2009). Impact of prenatal diagnosis on choledochal cysts and the benefits of early excision. J Paediatr Child Health.

[13] Ishibashi H, Shimada M, Kamisawa T, Fujii H, Hamada Y, Kubota M (2017). Japanese clinical practice guidelines for congenital biliary dilatation. J Hepatobiliary Pancreat Sci.

[14] Germani M, Liberto D, Elmo G, Lobos P, Ruiz E (2011). Choledochal cyst in pediatric patients: a 10-year single institution experience. Acta Gastroenterol Latinoam.

[15] Hanna DN, McKay KG, Ghani MO, Correa H, Zamora IJ, Lovvorn HN 3. Elective choledochal cyst excision is associated with improved postoperative outcomes in children. Pediatr Surg Int. 2022;38(6):817–24. 10.1007/s00383-022-05108-z 35338382

[16] Diao M, Li L, Cheng W (2012). Timing of surgery for prenatally diagnosed asymptomatic choledochal cysts: a prospective randomized study. J Pediatr Surg.

[17] Cochran ED, Lazow SP, Kim AG, Burkhalter LS, Frost NW, Stitelman D (2022). The in-utero diagnosis of choledochal cyst: can postnatal imaging predict benefit from early surgical intervention?. J Matern Fetal Neonatal Med.

[18] Guan X, Li J, Wang Z, Zeng J, Zhong W, Yu J. Timing of operation in children with a prenatal diagnosis of choledochal cyst: a single-center retrospective study. J Hepatobiliary Pancreat Sci. 2022. 10.1002/jhbp.1155 35435313

